# How Environment, Cognition, and Behavior Shape Doctoral Students’ Academic Career Intentions: Insights from a Comprehensive Study

**DOI:** 10.3390/bs15070990

**Published:** 2025-07-21

**Authors:** Wanhe Li, Xiaohan Jiang

**Affiliations:** School of Education, Tianjin University, Tianjin 300354, China; j_xiaohan@tju.edu.cn

**Keywords:** doctoral student, academic career intention, academic labor market, academic interest, career development path

## Abstract

Although career choice is a kind of individual behavior, as the gatekeeper of the discipline, doctoral students’ academic career intention reflects the attractiveness of the academic labor market and determines the sustainable development of academic careers. An analysis of data (N = 1322) from a survey among Chinese doctoral students reveals that (1) environmental factors, such as departmental atmosphere and advisor support, cognitive factors like academic interest and research self-efficacy, as well as behavioral factors including research engagement and publication rates, significantly promote doctoral students’ academic career intentions; (2) female doctoral students and those from prestigious institutions show stronger academic career aspirations; (3) the influence of interest factors on doctoral students’ commitment to an academic career is particularly pronounced, especially in the field of fundamental science; (4) a clear understanding of career paths positively moderates the effect of interest on academic career intentions. Within increasingly severe competition in the global academic labor market, it is necessary to provide more support for doctoral students who are willing to engage in academic careers by enhancing career planning guidance for doctoral students and supporting them in making rational career plans and adequate preparations.

## 1. Introduction

For a long time, the training and succession of future discipline “gatekeepers” has become a cultural cognitive schema within academic organizations in higher education, where doctoral students are viewed as the reserve personnel for academic professions. Doctoral education itself is regarded as a preparation process for academic professions. However, with the accelerated popularization of higher education globally, the scale of doctoral education continues to grow, and the supply and demand structure of the academic labor market is changing, profoundly impacting the career choices and development of doctoral students. Since the beginning of the 21st century, factors such as increasingly scarce tenured positions, the prevalence of short-term contracts, and intense competition for research resources have painted a bleak picture for the future academic careers of doctoral students (Cyranoski et al., 2019; Alberts et al., 2014; Liang & Li, 2021), creating a cooling effect on their academic career intentions (Bao et al., 2017). According to a survey by the National Science Foundation in the United States, the proportion of doctoral graduates entering academic professions in 2020 was 40%, a decrease of 9% compared to 2000. The lowest employment rate in academic positions was in the engineering field, at only 10%, followed by physics and earth sciences at 16%. With the exception of education, the proportion of doctoral graduates entering academic professions in other fields has declined over the past two decades (National Science Foundation, 2022). In China, since 2010, the number of doctoral graduates has begun to exceed the number of newly created full-time faculty positions. The overall supply of both Chinese and international doctoral graduates has increased rapidly, while the demand for academic positions in universities and research institutions has slowed down, leading to an overall surplus in the academic labor market. This trend is particularly evident in certain fields where supply significantly exceeds demand, resulting in a ‘surplus’ of doctoral graduates (Z. Li & Liang, 2021). Increasingly, graduates are applying for positions that do not require a doctorate, sparking heated discussions about whether ‘over education’ has begun.

In addition to changes in the supply and demand structure of the academic labor market, the transformation of knowledge production models and the professionalization process of professions have also led to increased demand for doctoral graduates in non-academic labor markets such as government departments and corporations. The ‘decoupling’ of doctoral education from academic careers (Shen et al., 2015) and the diversification of career development paths for doctoral graduates outside academia have already emerged in major countries with significant doctoral education, including China. The connotation and educational objectives of doctoral degrees need to be redefined, and the training process for doctoral students must be adjusted to meet diversified quality requirements (Barbara, 2007; Chen, 2010).

A century ago, Max Weber candidly stated in his lecture Science as a Vocation that ‘A young man who devotes himself to science as a career… it is extremely dangerous for a young scholar with no money to take any risks, given the conditions of an academic career… without having any idea of whether he will be able to find a reasonably paid job afterwards.’ (Weber, 2005). Today, the external temptations and constraints surrounding scientific research have become increasingly complex and pervasive. In China, the high demands and low salaries of academic careers, combined with the intensified workloads and pressures following appointment system reforms (Bao et al., 2017), seem to diminish the appeal of academic professions. Nevertheless, for many young individuals, the allure of an academic career remains a significant motivation for pursuing a doctorate.

So, what factors support doctoral students in engaging in this so-called ‘reckless gamble?’ Are they driven by ‘emotional impulses,’ as Weber suggested? Beyond emotional factors, what role does the cognition of academic careers play in this context? Existing studies have primarily focused on the influence of single-dimensional factors on doctoral students’ career intentions or merely examined their post-graduation destinations. However, few have systematically investigated the combined effects of environmental, cognitive, and behavioral factors on academic career intentions. Moreover, the moderating role of career development cognition in the relationship between academic interest and academic career intention remains underexplored, and the critical role of career planning in this process has yet to be fully elucidated. This study utilizes survey data from Chinese academic doctoral students to explore the influencing factors of their academic career intentions from three perspectives as follows: environmental, cognitive, and behavioral factors, focusing on the influencing mechanism of individual cognitive factors, especially the driving role of academic interest and the moderating effect of career awareness. This study primarily focuses on the following research questions: First, are doctoral students’ academic career intentions influenced by supportive environmental factors, such as departmental atmosphere, team support, and advisor support? Second, are these intentions shaped by individual cognitive factors, including academic interest and self-efficacy? In particular, does the effect of academic interest on academic career intention vary depending on the degree of career awareness? Third, do behavioral engagement factors, such as project, paper publications, and time investment, affect doctoral students’ academic career intentions? Fourth, what are the characteristics of career orientations and their influencing factors among doctoral students across different disciplinary backgrounds? By addressing these core questions, this study aims to enrich and extend theoretical understandings of academic career choices among doctoral students and systematically reveal the influencing factors. On a practical level, the findings will provide theoretical support for doctoral career development planning and offer evidence-based insights for universities and policymakers to improve academic training systems and enhance support for academic career development.

## 2. Literature Review

### 2.1. Environmental Factors and Doctoral Students’ Academic Career Intentions

Discussions regarding the academic career orientations of doctoral students often adopt the perspective of professional socialization theory. This theory indicates that the social interactions within teacher collectives and between students and teachers create a supportive culture, laying a strong foundation for the future academic careers of doctoral students (Weidman & Stein, 2003). This highlights the profound impact of environmental support on the academic careers of doctoral students. Once doctoral students pass their qualifying exams and become candidates, the initial phase of their academic careers begins, marking a crucial period for shaping and transforming their academic identities. The academic community must strategically provide support and help doctoral students clarify the responsibilities associated with academic professions (Foote, 2010). Empirical studies in China have also examined how organizational support, including academic career support and academic atmosphere, affects doctoral students’ academic competencies, professional identity, passion for academia, and career intentions (Y. Huang & Wang, 2020; Yang, 2020). In addition, some studies have further revealed significant heterogeneity in the influence of environmental support on doctoral students’ academic career intentions. Specifically, the positive effect of emotional support from supervisors and the negative effect of organizational emotional support are found to be significant only among high-ability doctoral students, whereas the positive effect of organizational academic career support is evident only among those with lower academic ability (Shao & Su, 2025).

Among the various environmental support factors, advisors serve as ‘significant others’ in the academic journeys of doctoral students (Bargar & Jane, 1983), exerting comprehensive influence on their research guidance and future career development. In particular, in China, advisors are viewed as the ‘primary responsible individuals’ for doctoral training, and providing multifaceted support is considered duty-bound for advisors and is essential to establishing a positive advisor–student relationship. Evidence suggests that the humanistic care provided by advisors significantly impacts the capability enhancement and socialization processes of doctoral students (W. Li & Li, 2021; L. Xu, 2020). Doctoral students who seek advice from their advisors and actively discuss career development when making career decisions are 1.2 and 1.3 times more likely to pursue academic careers compared to those who do not (N. Liu, 2021). Under the academic-oriented training goal, supervisors foster doctoral students’ academic interest through emotional interactions such as care, understanding, and encouragement (R. Wang & Zhong, 2023). In this study, organizational support is conceptualized along two dimensions: departmental atmosphere and team support.

### 2.2. Cognitive Factors and Doctoral Students’ Academic Career Intentions

Individual cognitive factors are generally regarded as primary motivators for career choice. According to the personality–job fit theory, individuals tend to select occupational environments that match their interests. This notion is frequently supported in academic career research. Tony Becher, in his work *Academic Tribes and Territories*, argues that the ability to autonomously choose research topics of interest is a major reason for pursuing and committing to an academic career (Tony & Paul, 2008). Interest helps doctoral students maintain their academic engagement and perseverance (Finch et al., 2013); those displaying high interest and enthusiasm for academia often exhibit more pronounced innovative capabilities (Ma et al., 2019) and a greater inclination toward academic careers (Y. Zhao & Hong, 2014). However, survey analyses indicate that although up to 80% of students express interest in academic careers at the outset of their doctoral studies, 25% report a decline in this interest by the time of graduation. This decline may stem from changes in personal career preferences or self-perceptions regarding research abilities (Roach & Sauermann, 2017), as well as the intensifying competition within the academic labor market (Bao et al., 2017). Regardless of whether the reasons are personal or market-related, doctoral students experience a comprehensive reflection on their capabilities, leading to perceptual judgments about their suitability for academic careers, which encapsulates the concept of self-efficacy. Consequently, self-efficacy is often considered closely related to academic career identity (Wu & Zhao, 2021). Existing research has demonstrated that doctoral students’ perceived improvement in academic competence, reflected in increased self-efficacy, significantly enhances their intention to pursue academic careers (Luo et al., 2021). Although the moderating role of career awareness in the relationship between academic interest and academic career intention has not been directly confirmed, it is recognized as an essential component of career self-efficacy and has been shown to moderate the effect of supervisor support on the academic career development of doctoral students (Hou et al., 2025). Moreover, advisor support has been found to promote doctoral students’ academic career socialization by reinforcing their awareness of career planning (X. Zhang & Yang, 2024).

### 2.3. Behavioral Factors and Doctoral Students’ Academic Career Intentions

Throughout their academic journeys, participation in scholarly activities can assist doctoral students in learning from and emulating the behaviors of community members, clarifying role norms to guide their own practices. This deep involvement can enhance their understanding and identification with their profession. Consequently, doctoral students with rich research experiences may develop stronger intentions toward academic careers. Research has shown that insufficient time invested in research activities increases the likelihood of doctoral students failing to obtain their degrees and decreases their sense of identification with academic careers (Martinsuo & Turkulainen, 2011). Additionally, academic publications significantly impact a doctoral student’s chances of securing positions in academia, with those who publish more papers being more inclined to work in universities or research institutions (Z. Xu, 2018). In addition, studies have found that the number of paper publications during doctoral studies, as well as the accumulation of academic career experiences such as independently teaching courses and applying for research projects, significantly increases doctoral students’ intentions to pursue academic careers (Luo et al., 2021).

### 2.4. Individual Background Factors and Doctoral Students’ Academic Career Intentions

Numerous studies, both within China and internationally, have explored the relationship between individual background factors and doctoral career orientations. Limited attention has been paid to whether academic career intentions differ significantly among doctoral students across disciplinary backgrounds. Most existing studies have instead focused on differences in post-graduation outcomes. For instance, a study based on data from Shanghai Jiao Tong University examined the career choices of doctoral graduates in engineering, natural sciences, humanities and social sciences, life sciences, and medicine. The results indicated that graduates in the humanities and social sciences were most likely to pursue academic careers, followed by those in the natural sciences and life sciences, with lower proportions in engineering and the lowest in medicine (Wei et al., 2021). Other studies have concentrated on specific disciplinary categories, such as basic research fields. One such study found a rapid increase in the proportion of basic research doctoral graduates entering academic careers. However, within these fields, graduates in the natural sciences and engineering were more likely to pursue non-academic careers compared to those in other disciplines (Xiang et al., 2022).

This study includes doctoral students’ gender, year of study, institutional reputation, and enrollment method as control variables, as these factors have been shown in previous research to be associated with academic career intentions. Firstly, regarding gender, some studies also suggest that women prefer more stable academic careers or are inclined to work in universities (Y. Zhao & Hong, 2014; Roach & Sauermann, 2017; Wu & Zhao, 2021; Martinsuo & Turkulainen, 2011). Conversely, other research indicates that men show significantly higher interest in academic careers (Roach & Sauermann, 2017) and a greater likelihood of pursuing them (Yang, 2020), while some findings suggest that gender may not significantly impact these factors (C. Jiang, 2011). These factors identified in prior research will be used as control variables in this study. Thirdly, with respect to institutional reputation, according to screening hypothesis theory, doctoral graduates from more prestigious institutions are more likely to have their abilities recognized in the job market based on the educational signals they provide (Z. Li & Liang, 2021). Discussions regarding the impact of institutional reputation on academic careers highlight the entrenched hierarchical structure within the academic labor market. Numerous studies internationally indicate that graduates from the highest-ranking institutions are more likely to be hired by similarly ranked institutions (Crane, 1970; Hargens & Hagstrom, 1967). A study focusing on the cross-institutional hiring network in the field of business administration found that doctoral graduates are predominantly recruited by academic units from “Project 985” universities and overseas institutions. The findings also revealed a clear hierarchical stratification in doctoral employment across universities, characterized by a general pattern of “downward mobility,” in which most doctoral graduates ultimately secure positions at institutions ranked lower than their alma maters (H. Jiang et al., 2023). Some studies by Chinese scholars on doctoral graduates in physics and chemistry have also confirmed the existence of hierarchical structures in hiring networks (B. Zhang, 2013; L. Li & Shen, 2019). Therefore, it is reasonable to hypothesize that doctoral graduates from higher-reputation institutions have more employment opportunities and stronger academic career intentions. Finally, with regard to enrollment method, doctoral students admitted through the standard entrance examination are most likely to be driven by intrinsic motivation, primarily reflected in a strong interest in academic research and a clear intention to pursue an academic career. In contrast, those admitted directly from undergraduate programs tend to be more influenced by extrinsic motivation, while those in integrated master–doctoral programs are most commonly motivated by a combination of intrinsic and extrinsic factors (H. Huang & Jin, 2016).

Overall, as issues such as budget cuts in universities, deteriorating working conditions for faculties, and the instability and uncertainty of career development paths proliferate globally, discussions regarding the academic career crisis have become increasingly common (Bao et al., 2017). In this context, the focus of academic career research has gradually shifted from faculty and researchers to the doctoral student population. To comprehensively examine the factors influencing doctoral students’ academic career intentions, this study will explore the effects of environmental support, individual cognition, and behavioral engagement on these intentions.

## 3. Materials and Methods

### 3.1. Data

This study utilizes data from a survey on the research experiences of academic doctoral students. The survey employed a convenience sampling method after taking into account both the types of universities (including “Project 985” universities, “Project 211” universities, and other institutions) and their regional distribution across eastern, central, and western China. A total of 23 universities were selected for online questionnaire distribution. These included eight “Project 985” universities: Tsinghua University, Beihang University, Hunan University, Nanjing University, Shanghai Jiao Tong University, Tianjin University, Wuhan University, and Northwest A&F University; three “Project 211” universities: Nanjing Normal University, Soochow University, and Southwest University; and twelve other institutions: University of Chinese Academy of Sciences, Peking Union Medical College, Chinese Academy of Social Sciences, Dongbei University of Finance and Economics, South China Agricultural University, Ningxia Medical University, Qinghai University, Tarim University, Changchun University of Chinese Medicine, among others. A total of 1322 valid questionnaires are collected in this survey. In terms of gender distribution, 688 respondents are male, accounting for 52.0% of the sample, while 634 are female, making up 48.0%. Regarding disciplinary distribution, the sample includes Literature (32, 2.4%), History (21, 1.6%), Philosophy (20, 1.5%), Education (37, 2.8%), Economics (40, 3.0%), Law (42, 3.2%), Science (231, 17.5%), Engineering (490, 37.1%), Agriculture (186, 14.1%), Medicine (138, 10.4%), Management (80, 6.1%), and Arts (5, 0.3%). The gender and disciplinary distribution of the survey sample closely aligns with national statistics on doctoral education in China, indicating good representativeness.

### 3.2. Variables

The dependent variable in this study is doctoral students’ academic career intentions, measured through the item “I hope to engage in research-related professions after obtaining my degree.” This intention is assessed on a 5-point Likert scale. Descriptive statistics reveal that the average score for doctoral students’ academic career intentions is 3.91. If we consider those who select “agree” or “strongly agree” as inclined toward an academic career, approximately 69.7% of doctoral students show a tendency to pursue academic careers after graduation. The core independent variables encompass three aspects as follows: environmental support, individual cognition, and behavioral engagement. The basic conditions and measurement descriptions of all variables are summarized in Table 1.

## 4. Results

### 4.1. Common Method Bias Test

As the data in this study were collected through self-reports from the same respondents, there may be a risk of common method bias among the variables. Following the approach of previous research (Zhou & Long, 2004), Harman’s single-factor test was conducted to assess potential bias. An exploratory factor analysis was performed using principal component analysis on all measurement items in the scales. Four factors were extracted, and the first factor accounted for 35.13% of the total variance, which is below the critical threshold of 40%. This result suggests that common method bias is not a serious concern in this study.

### 4.2. Test of Model Appropriateness

Before conducting the formal regression analysis, this study first tested whether a significant nested data structure existed. Following previous research (Yu et al., 2021), a one-way analysis of variance (ANOVA) was conducted on academic career intention scores, and the intraclass correlation coefficient (ICC) was subsequently calculated. According to Cohen et al.’s (1983) guideline, multilevel regression models are recommended when ICC values are equal to or greater than 0.059. The analysis showed that the ICC across 23 institutions was approximately 0.0077, which is far below the recommended threshold. This indicates low within-group correlation and minimal between-group variance. Therefore, the subsequent analysis adopts hierarchical regression models at the individual level, without the need for hierarchical linear modeling (HLM).

### 4.3. Analysis of Influencing Factors for Doctoral Students’ Academic Career Intentions

Hierarchical linear regression was employed to analyze the influencing factors for doctoral students’ academic career intentions, with results presented in Table 2. Model 1 serves as the baseline model, which includes only control variables. The results indicate that the year of study significantly impacts doctoral students’ academic career intentions. This aligns with previous research findings that show while doctoral students initially have a strong interest in academic careers, the proportion of those who maintain this strong interest decreases noticeably after three and a half years (Roach & Sauermann, 2017). Additionally, compared to students enrolled in a combined bachelor’s and doctoral degree, those enrolled through regular admission and successive master–doctor programs show significantly higher academic career intentions. This supports earlier research suggesting that doctoral students admitted through exam-based and application-review methods are more likely to maintain their commitment to academic careers before and after enrollment (Kuang & Li, 2019), whereas those enrolled in a combined bachelor’s and doctoral degree may experience a decline in academic enthusiasm (Hong et al., 2022). From the adjusted R^2^ value, it is evident that the overall explanatory power of the model is only 1.8%. Therefore, it is necessary to introduce key independent variables to enhance the explanatory power of the model.

- The standardized residual scatter plot, histogram with a normal curve, and P-P plot indicate homoscedasticity and approximate normal distribution of residuals. The DW test value is 1.977, suggesting that observations are independent of each other.
- The multicollinearity test results show VIF values below 3, indicating no severe multicollinearity issues among independent variables.

Building on Model 1, Model 2 incorporates variables related to doctoral students’ behavioral engagement. The results indicate that the longer doctoral students invest time in research activities, the stronger their desire to pursue academic-related careers. Additionally, the number of published papers significantly enhances doctoral students’ academic career aspirations. However, the adjusted R^2^ value reveals that the overall explanatory power of Model 2 remains below 3%, indicating that the overall explanatory strength is still limited and that further inclusion of core independent variables is warranted.

Furthermore, adding variables related to environmental support yields the regression results for Model 3. The newly included variables—departmental atmosphere, team support, and advisor support—demonstrate a significant positive influence. Consequently, the overall explanatory power of Model 3 increases to 24.2%. This strongly supports the views associated with professional socialization theory, which posits that departments, advisors, peers, and other faculty members all play a role in influencing doctoral students’ transition to becoming independent scholars (Lovitts, 2008). The academic atmosphere, peer relationships, and student–faculty interactions that collectively constitute the academic organizational culture encourage doctoral students to deepen their understanding of the academic community (Weidman et al., 2001), which may in turn contribute to their identification with and pursuit of an academic career path.

Model 4 represents the overall results concerning the factors influencing doctoral students’ academic career intentions, with explanatory power increasing to 52.5% compared to Models 1 to 3. The findings indicate that gender has a significant impact on academic career aspirations, with female doctoral students exhibiting a stronger desire to pursue academic careers than their male counterparts. Among the institutional reputation variables, ‘Project 985’ and ‘Project 211’ universities (both of them are key university construction projects initiated by the Ministry of Education of China and represent high reputation institutions among Chinese universities) positively influence academic career intentions, although only the effect of ‘Project 211’ universities reaches statistical significance. The enrollment method variables, departmental atmosphere and advisor support still exert significant positive effects on doctoral students’ academic career intentions. In the newly introduced individual cognitive variables, both academic interest and research self-efficacy significantly enhance doctoral students’ academic career intentions. Notably, the regression coefficient for academic interest is as high as 0.575, indicating that doctoral students’ intentions to pursue academic careers are, to a certain extent, driven by their academic interests.

To examine the moderating effect of career awareness on the relationship between academic interest and academic career intentions, Model 5 incorporates the product term of academic interest and career awareness based on Model 4. The results indicate that career awareness significantly positively moderates the impact of academic interest on academic career intentions. However, survey results reveal that the clarity of career awareness among doctoral students is relatively low, with only 58.1% feeling clear about their career pathways. As some studies have pointed out, while doctoral students may be relatively well-prepared for academic research and confident in their research abilities, they often lack adequate awareness and preparation for other aspects of academic careers, such as teaching, mentoring students, management, evaluation, and social service. Additionally, the ethical responsibilities of academic professions are not effectively conveyed to the next generation of practitioners (Y. Zhang, 2009; Shen et al., 2022).

### 4.4. Academic Career Intentions of Doctoral Students in Different Disciplines and Their Influencing Factors

**Figure 1:**
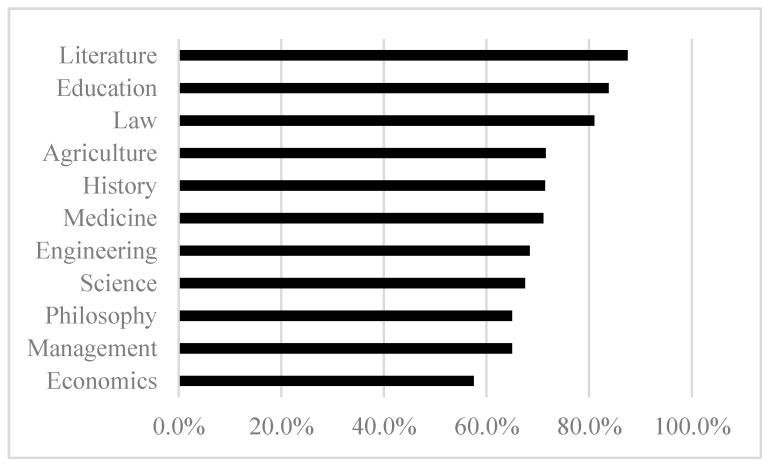
Academic career intentions of doctoral students by discipline type.

Different academic disciplines have unique knowledge attributes and research paradigms, leading to varying patterns of research development and talent cultivation (Gu & Lin, 2011). As shown in Figure 1, doctoral students’ willingness to pursue research-related careers differs significantly across disciplines, ranging from less than 60% to nearly 90%. Specifically, fields like Literature (87.5%), Education (83.8%), and Law (81.0%) exhibit the highest levels of academic career intentions, all exceeding 80%. The second tier includes disciplines such as Agriculture (71.5%), History (71.4%), Medicine (71.1%), Engineering (68.4%), and Science (67.5%). The third tier, consists of Philosophy (65.0%), Management (65.0%), and Economics (57.5%).

Next, a grouped regression analysis is conducted to further explain the characteristics of factors influencing the academic career intentions of doctoral students across different disciplinary types. Based on the ‘Strengthening Foundation Plan’ (a policy in China aimed at strengthening the cultivation of talent in fundamental disciplines), we categorize disciplines such as Science, History, Philosophy, and Literature as fundamental disciplines. Other disciplines are grouped into ‘Applied Disciplines-Natural’, which includes Engineering, Agriculture, and Medicine, and ‘Applied Disciplines-Social’, which encompasses Economics, Law, Management, and Education. This results in three disciplinary types for the grouped regression analysis. The results are shown in Table 3. After controlling for background variables, academic interest continues to have a significant positive effect on doctoral students’ academic career intentions, and this conclusion remains robust. Notably, in the fundamental disciplines group, the regression coefficient for academic interest exceeds 0.8, indicating a significantly greater influence compared to the other two groups. This further highlights the crucial role that the factor of interest plays in motivating doctoral students in fundamental disciplines to pursue an academic career.

The results regarding the impact of environmental support exhibit certain heterogeneity, reflecting the organizational patterns of research activities and talent cultivation characteristics across different academic fields. In fundamental disciplines, the incremental and structured approaches to knowledge accumulation often require individuals to engage in long-term exploration of specific problems in pursuit of breakthroughs. As a result, the perceived environmental support for doctoral students is more likely to come from their advisors, making advisor support particularly significant for their academic career intentions.

In the applied sciences within the natural sciences, research activities are more often conducted through organizational forms such as laboratories, and mentorship frequently manifests as collective guidance in a one-to-many setting. Consequently, the professional socialization of doctoral students appears to be increasingly shaped by the broader research team, with team support potentially emerging as the most influential factor in their development.

In contrast, within the applied disciplines of the social sciences, although academic activities have increasingly been conducted in the form of research groups in recent years, the research training process for doctoral students still bears the characteristics of a traditional mentor–apprentice relationship. At the same time, it heavily relies on various professional academic activities, including lectures, seminars, salons, workshops, including interdisciplinary exchanges for inspiration. Therefore, both the academic atmosphere of the department and advisor support have significant impacts.

## 5. Discussion

First, the departmental atmosphere and advisor support have a significant positive impact on doctoral students’ academic career intentions. If doctoral students perceive a strong research atmosphere within their department that motivates their scientific work during their academic training, they are more likely to aspire to academic careers. As emphasized in the theory of doctoral student socialization, the research atmosphere serves as an organizational culture that is a crucial influencing factor in the development of doctoral students into industry novices with professional commitment and identity. Advisors, as role models for doctoral students and representatives of the departmental organization, play the most critical role in the socialization process, establishing standards and norms for their behaviors (Girves & Wemmerus, 1988). If advisors provide conditions and opportunities, supporting and guiding doctoral students to integrate into the academic community, it will significantly promote their academic career development. In China, where public universities dominate the higher education landscape, academic evaluations at the departmental level, assessments of supervisors’ research performance, and corresponding funding mechanisms are all heavily influenced by administrative authorities. In the context of global scientific and technological competition, the government has introduced a series of science and education policies that place strong emphasis on research outputs and project performance in higher education institutions. As a result, academic departments and supervisors are increasingly inclined to provide doctoral students with extensive research training and support, laying a solid foundation for enhancing their academic career intentions. However, there is also a growing concern that instrumental rationality may become an implicit standard governing educational practices and human development (Z. Zhang, 2021), which may contribute to a “performance paradox” among supervisors, universities, and the higher education system as a whole. Academic climate and support culture, as elements of a long-term-oriented soft environment, have been argued by some scholars to exert a more profound influence on doctoral students’ creativity and academic character than institutional or material conditions (L. Liu et al., 2023; He et al., 2025). An inclusive and trial-tolerant supportive environment is more likely to help doctoral students perceive and internalize academic values, thereby motivating them to pursue academic careers.

Second, the significant promoting effects of academic interest and research self-efficacy on doctoral students’ academic career intentions have been confirmed, and they possess high explanatory power in the regression model. Notably, the effect of academic interest is particularly pronounced and remains robust in the grouped regression analysis. This indicates that doctoral students are indeed driven by ‘emotional impulses’ (Weber, 2005) when preparing to enter academic careers; this ‘unique enthusiasm and passion’ is a prerequisite for developing scientific aspirations. However, academic career intentions are not solely based on pure emotion, appearing completely reckless and uninformed; they are also influenced by self-efficacy judgments and moderated by clarity in career development pathways. This suggests that if doctoral students lack awareness of the academic field and its associated risks, basing their aspirations solely on self-imagined notions and others’ words (X. Zhang, 2022), they may easily lose their original ideals of truth-seeking and innovation in scientific research under real-world challenges, diminishing their desire for academic careers. Especially after the emergence of a ‘turning point’ in the supply side of doctoral education in China, the elite academic labor market represented by research universities is becoming increasingly saturated, which may contribute to heightened entry requirements for doctoral students and intensified competition for academic positions. Consequently, more doctoral graduates seeking faculty positions will find themselves transitioning into regional teaching-research or teaching-focused universities (S. Zhao & Shen, 2010). There is an urgent need to enhance career planning guidance for doctoral students, helping them recognize changes in the academic labor market, understand the content, characteristics, and required competencies of different types of academic careers, and make rational career plans and adequate preparations.

Third, the more time doctoral students invest in research activities and the more papers they publish, the stronger their academic career intentions tend to be. This is particularly relevant to the current high expectations for paper publication in Chinese higher education institutions. Writing and publishing academic papers is one of the essential skills that doctoral students must master during their research training and is a prerequisite for recognition and acceptance by the academic community, serving as an essential pathway into the academic field. As the senior academic job market becomes increasingly saturated, the ‘entrance fee’ that doctoral students must pay to enter the academic field is becoming more expensive (Yuan, 2012). Graduates with more publications have greater bargaining power in the academic labor market and consequently more opportunities to enter academic careers. Therefore, the positive relationship between doctoral students’ research output, time investment in research, and their academic career intentions reflects not only individual effort but is also shaped by the combined influences of policy mechanisms and market structures. In the context of an increasingly competitive academic labor market and growing concentration of academic resources, it is essential to advance reform of the academic evaluation system at the policy level. Such reform should aim to reduce the excessive pressure on doctoral students to publish and promote more diverse and sustainable academic career pathways.

Fourth, the results of grouped regression analysis on the influencing factors of academic career intentions among doctoral students in different disciplines indicate that, in the field of fundamental disciplines, interest plays a particularly prominent role in motivating doctoral students to pursue academic careers, and support from advisors significantly enhances their inclination to choose academic professions. Intellectual interest in academia is the starting point of an academic career and serves as the driving force behind doctoral students’ exploration of academic paths. In light of current policies promoting talent cultivation in STEM fields and fundamental research in various countries, it is essential to cultivate and maintain academic interest, foster a positive research ecological culture, and attract more students who genuinely love scientific endeavors to engage deeply and innovate courageously. Due to their long research cycles, slow translation of results, and limited short-term returns, fundamental disciplines often lack direct market incentives. As a result, compared to applied disciplines, academic career choices in these fields rely more heavily on doctoral students’ intrinsic passion for and commitment to academic research. From a national strategic perspective, fundamental disciplines serve as the foundation of a country’s capacity for original innovation and strategic scientific advancement. In response to the intensifying global competition in science and technology, many countries have introduced policies to strengthen the cultivation of talent in STEM fields and basic research, aiming to build and stabilize a cohort of young scholars with strong research potential and sustained academic interest. Therefore, strengthening interest-driven mechanisms, improving supervisory support systems, and fostering a robust research culture are not only critical for enhancing doctoral students’ academic career intentions but also essential for reinforcing the national reserve of scientific and technological innovation capacity.

Finally, the examination of control variables reveals that female doctoral students have a stronger intention to pursue academic careers. Over the past two decades, the proportion of female doctoral students in China has significantly increased, rising from 18.52% in 1997 to 41.87% in 2020, reflecting substantial progress in gender equality and educational advancement. Nonetheless, numerous studies indicate that female doctoral students’ academic experiences and backgrounds are significantly lower than those of their male counterparts, and this situation extends into academic careers post-graduation (W. Wang et al., 2021). On the one hand, longstanding social role divisions and implicit gender bias have contributed to the persistent issue of the “leaky pipeline,” whereby women face greater challenges in achieving higher levels of success in academic careers. These structural gender barriers are not merely the result of individual choices, but are deeply embedded in academic organizational cultures, research evaluation systems, and policy environments, reflecting the tension between institutional structures and gender norms. On the other hand, from an economic perspective, the high demands and uncertain returns associated with academic careers can impose additional constraints on women, particularly in the absence of gender-sensitive support policies. Therefore, although female doctoral students often express stronger intentions to pursue academic careers, whether they can obtain sufficient institutional and cultural support remains a critical issue that the current academic system must urgently address. Additionally, institutional reputation has a significant impact on doctoral students’ academic career intentions. Compared to other universities, doctoral students from ‘Project 211’ universities exhibit a stronger desire for academic careers. Although the data for doctoral students from ‘Project 985’ universities did not reach statistically significant levels, their academic career intentions are still higher than those from other reference institutions. This finding supports existing research (C. Li et al., 2019). Given that key universities possess stronger research platforms, faculty resources, and a more robust research atmosphere, these factors may contribute to a stronger inclination for doctoral students to pursue academic careers. On the one hand, research-oriented universities prioritized in national development strategies shoulder greater responsibilities in scientific research and talent cultivation, benefiting from preferential policy support and resource allocation. These structural advantages position them at the center of China’s science and technology strategy and higher education reform and may influence their doctoral students’ identification with and expectations for academic careers. On the other hand, such institutions generally possess stronger research capacities, more stable funding, and broader academic networks. These factors not only enhance doctoral students’ research productivity but also boost their confidence and motivation to pursue academic careers, thereby fostering stronger career intentions. Taken together, both gender differences and institutional disparities reflect the combined influence of systemic structures, resource distribution, and academic culture. Promoting a more diverse, equitable, and sustainable pathway to academic careers for doctoral students requires continued reform of research governance and optimization of resource allocation across institutions.

## 6. Conclusions

Based on empirical analysis of data from a survey on the research experiences of academic-track doctoral students, this study yields the following key findings: first, departmental climate and supervisor support have significant positive effects on doctoral students’ academic career intentions, indicating that supportive environmental factors—particularly academic atmosphere at the organizational level and support from key individuals—play a crucial role in shaping and strengthening academic career aspirations. Second, academic interest and research self-efficacy significantly promote academic career intentions, suggesting that individual cognitive factors are important psychological mechanisms influencing career orientation. Moreover, career development cognition exerts a significant moderating effect on the relationship between academic interest and academic career intention; that is, the clearer the doctoral students’ rational understanding of academic career pathways, the stronger the motivating effect of academic interest. Third, greater time investment in research activities and a higher number of publications are associated with stronger academic career intentions, highlighting the positive role of research-related behaviors in shaping career preferences. Fourth, compared to applied disciplines, academic interest plays a more prominent role in driving academic career intentions among doctoral students in fundamental disciplines. Additionally, female doctoral students demonstrate stronger academic career intentions than their male counterparts, and affiliation with institutions of higher organizational reputation is associated with enhanced academic career intentions. These results point to the complex interactive effects of individual background characteristics on career orientation. In summary, this study develops an integrated analytical framework based on the interaction of environmental, cognitive, and behavioral factors to explain doctoral students’ academic career intentions. It deepens the systematic understanding of how supportive environments, individual cognition, and behavioral engagement jointly influence academic career development. The identified moderating role of career development cognition in the link between academic interest and career intention also enriches the explanatory pathways of academic career formation. Furthermore, the study provides empirical and theoretical insights into the career decision-making processes of doctoral students with diverse backgrounds and offers a basis for more targeted and differentiated interventions in future career guidance.

The career choices of doctoral students are driven by interests and rational self-assessment, representing a balancing and decision-making process among multiple needs and expectations, such as work environment, economic benefits, and self-actualization. While career choice is an individual behavior, the phenomenon of talented doctoral graduates leaving academia and the exodus of academic elites warrants attention, as it reflects a decline in the attractiveness of academic careers. China’s academic labor market is an imperfect market in which the supply, demand, and compensation structures are heavily dependent on government regulation. Therefore, examining academic careers and doctoral students’ career choices holds significant policy and practical value. At the policy-making level, efforts should be directed toward optimizing career development pathways for postdoctoral researchers and early-career scholars, improving institutional guarantees and compensation systems for academic careers, and strengthening doctoral students’ confidence in pursuing academic trajectories. At the institutional management level, universities should prioritize supervisor support and the cultivation of a positive academic climate within departments, while also enhancing career development guidance and training programs for doctoral students. Through coordinated efforts between policy frameworks and university practices, it is possible to enhance the attractiveness of academic careers and improve talent retention. This, in turn, will contribute to strengthening China’s reserve of high-level academic talent and fostering a virtuous cycle within the national research and innovation system.

## 7. Limitations

In terms of sampling strategy, although the use of convenience sampling ensured the feasibility of this study, it may limit the generalizability of the findings. Future research should consider adopting stratified sampling to improve the representativeness of the sample. In addition, during questionnaire distribution, more detailed background information—such as respondents’ academic departments and disciplines—should be collected, so that future analyses can more accurately determine whether a hierarchical linear modeling approach is appropriate. Due to data limitations, this paper only discusses the effects of environmental support, individual cognition, and behaviors of doctoral students, without incorporating control variables like family socioeconomic status, nor does it delve into the specific impact of labor market factors related to academic career conditions on doctoral students’ academic career choices. These areas will be the focus of ongoing research in the future. It is particularly important to note that doctoral students are often subject to institutional constraints during admission, training, and promotion processes. These external structural factors interact with students’ subjective experiences of academic career development, yet such dynamics remain underexplored in existing research. Future studies should incorporate variables related to the institutional and policy environment, as well as academic career entry standards, and adopt longitudinal approaches to more thoroughly examine the interaction between external structures and individual career intentions. This would allow for a more comprehensive understanding of the underlying logic and influencing mechanisms shaping doctoral students’ academic career choices.

## Figures and Tables

**Table 1:** Variable characteristics and measurement descriptions.

	Variable	M	SD	Measurement
Dependent Variable	Academic Career Intention	3.91	1.07	I hope to engage in research-related professions after obtaining my degree
Control Variables (Background Characteristics)	Gender	—	—	0 for female, 1 for male
	Year of Study	—	—	1 for first year, 2 for second year, 3 for third year, 4 for fourth year and above
	Institutional Reputation	—	—	1 for “Project 985” universities, 2 for “Project 211” universities, 3 for other universities
	Enrollment Method	—	—	1 for regular admission, 2 for a successive master–doctor program, and 3 for joined study of bachelor and doctoral degree
Independent Variables (Environmental Support)	Departmental Atmosphere	3.67	1.15	The research atmosphere of the department motivates my research work
	Team Support	3.89	1.10	The research team (project group/laboratory/mentorship group, etc.) can effectively support my research work
	Advisor Support	3.96	1.15	My advisor helps me integrate into the academic community
Independent Variables (Individual Cognition)	Academic Interest	3.89	0.99	I am very interested in participating in research activities
	Self-Efficacy	3.91	0.98	I am confident I can succeed in research activities
Independent Variables (Behavioral Engagement)	Project Participation	—	—	0 for not participated, 1 for participated
	Paper Publications	2.4	3.27	Number of journal articles and conference papers published
	Time Investment	44.75	21.95	Hours per week dedicated to research activities (e.g., literature review, surveys, experiments, group meetings, writing, academic discussions)
Moderating Variable	Career Awareness	3.64	1.097	I feel clear about my future career development path

**Table 2:** Regression analysis results of influencing factors for academic career intentions of doctoral students.

	Variable	Model 1	Model 2	Model 3	Model 4	Model 5
Background Variables	Gender	−0.030 (0.059)	−0.043 (0.059)	0.027 (0.053)	−0.034 * (0.042)	−0.034 * (0.042)
	Year (1st year as reference)					
	2nd Year	−0.062 ** (0.075)	−0.077 ** (0.076)	−0.009 (0.068)	0.027 (0.054)	0.024 (0.053)
	3rd Year	−0.078 *** (0.082)	−0.105 *** (0.083)	−0.034 (0.074)	0.015 (0.059)	0.007 (0.059)
	4th Year and Above	−0.030 (0.091)	−0.068 ** (0.094)	−0.010 (0.084)	0.021 (0.067)	0.008 (0.066)
	School Level (other universities as reference)					
	‘Project 985’ Universities	0.011 (0.099)	0.004 (0.099)	0.034 (0.088)	0.028 (0.070)	0.036 (0.069)
	‘Project 211’ Universities	0.056 (0.131)	0.045 (0.131)	0.061 (0.116)	0.065 ** (0.092)	0.069 *** (0.091)
	Enrollment Method (joined study of bachelor and doctoral degree as reference)					
	Regular admission	0.155 *** (0.100)	0.153 *** (0.100)	0.153 *** (0.088)	0.060 ** (0.070)	0.060 ** (0.070)
	Successive master–doctor program	0.069 * (0.113)	0.054 (0.114)	0.061 * (0.114)	0.034 (0.080)	0.034 (0.079)
Behavioral Engagement	Project Participation		−0.012 (0.076)	−0.008 (0.067)	−0.016 (0.053)	−0.020 (0.053)
	Paper Publications		0.103 *** (0.009)	0.083 *** (0.008)	0.032 (0.007)	0.026 (0.007)
	Time Investment		0.060 ** (0.001)	0.042 * (0.001)	0.023 (0.001)	0.027 (0.001)
Environmental Support	Departmental Atmosphere			0.202 *** (0.032)	0.063 ** (0.026)	0.046 * (0.026)
	Team Support			0.214 *** (0.036)	0.034 (0.029)	0.033 (0.029)
	Advisor Support			0.121 *** (0.033)	0.055 * (0.027)	0.065 ** (0.027)
Individual Cognition	Academic Interest				0.575 *** (0.029)	0.436 *** (0.040)
	Research Self-efficacy				0.174 *** (0.028)	0.145 *** (0.028)
	Academic Interest × Career Awareness					0.203 *** (0.006)
Constant		3.701 *** (0.0141)	3.545 *** (0.155)	1.438 *** (0.177)	0.371 ** (0.151)	0.721 *** (0.159)
F-value		3.950 ***	4.557 ***	30.935 ***	91.862 ***	89.890 ***
Adjusted R2		0.018	0.029	0.242	0.525	0.535

Note: *** indicates *p* < 0.01, ** indicates *p* < 0.05, and * indicates *p* < 0.1; values in parentheses are standard errors.

**Table 3:** Regression analysis results of influencing factors for academic career intentions of doctoral students by discipline type.

		Fundamental Disciplines	Applied Disciplines-Natural	Applied Disciplines-Social
Behavioral Engagement	Paper Publications	0.043 (0.016)	0.012 (0.008)	0.087 (0.018)
	Time Investment	0.002 (0.002)	0.027 (0.001)	0.116 ** (0.003)
	Project Participation	−0.039 (0.091)	−0.001 (0.073)	−0.032 (0.151)
Environmental Support	Departmental Atmosphere	0.008 (0.055)	0.056 (0.032)	0.104 * (0.058)
	Team Support	0.014 (0.052)	0.067 * (0.040)	0.017 (0.075
	Advisor Support	0.094 * (0.051)	0.013 (0.037)	0.127 * (0.062)
Emotional Cognition	Academic Interest	0.803 *** (0.062)	0.517 *** (0.037)	0.504 *** (0.078)
	Research Self-efficacy	0.019 (0.056)	0.199 *** (0.037)	0.176 *** (0.070)
Control Variables		Yes	Yes	Yes
Constant		0.757 * (0.424)	0.916 *** (0.914)	0.961 (0.914)
F-value		29.494 ***	58.188 ***	9.798 ***
Adjusted R2		0.602	0.531	0.416
Sample Size		302	808	199

Note: *** indicates *p* < 0.01, ** indicates *p* < 0.05, and * indicates *p* < 0.1; values in parentheses are standard errors.

## Data Availability

The data presented in this study is available on request from the corresponding author.
